# Cytokines and Growth Factors Promote Airway Smooth Muscle Cell Proliferation

**DOI:** 10.5402/2012/731472

**Published:** 2012-07-08

**Authors:** R. Stamatiou, E. Paraskeva, K. Gourgoulianis, P.-A. Molyvdas, A. Hatziefthimiou

**Affiliations:** ^1^Department of Physiology, Faculty of Medicine, University of Thessaly, Biopolis, 41110 Larissa, Greece; ^2^Department of Respiratory Medicine, Faculty of Medicine, University of Thessaly, 41110 Larissa, Greece

## Abstract

Chronic airway diseases, such as asthma or chronic obstructive pulmonary disease, are characterized by the presence in the airways of inflammation factors, growth factors and cytokines, which promote airway wall remodelling. The aim of this study was to investigate the effect of cytokines and growth factors on airway smooth muscle cell (ASMC) proliferation, phenotype and responsiveness. Incubation of serum starved human bronchial ASMCs with TNF-**α**, TGF, bFGF, and PDGF, but not IL-1**β**, increased methyl-[^3^H]thymidine incorporation and cell number, mediated by the PI3K and MAPK signalling pathways. Regarding rabbit tracheal ASMC proliferation, TNF-**α**, IL-1**β**, TGF, and PDGF increased methyl-[^3^H]thymidine incorporation in a PI3K- and MAPK-dependent manner. bFGF increased both methyl-[^3^H]thymidine incorporation and cell number. Moreover, incubation with TGF, bFGF and PDGF appears to drive human ASMCs towards a synthetic phenotype, as shown by the reduction of the percentage of cells expressing SM-**α** actin. In addition, the responsiveness of epithelium-denuded rabbit tracheal strips to carbachol was not significantly altered after 3-day treatment with bFGF. In conclusion, all the tested cytokines and growth factors increased ASMC proliferation to a different degree, depending on the specific cell type, with bronchial ASMCs being more prone to proliferation than tracheal ASMCs.

## 1. Introduction

Inflammatory airway diseases, like asthma and chronic obstructive pulmonary disease (COPD), despite their different pathophysiological process exhibit some common features: remodelling of the airway wall [1] and bronchial hyperresponsiveness to diverse stimuli. Airway remodelling, in particular, comprises structural changes of airways such as smooth muscle hypertrophy and hyperplasia, thickening and fibrosis of subepithelial basement membrane, hypertrophy of bronchial glands, goblet cell hyperplasia, and thickening of airway epithelium [1]. A tight interaction between inflammation and airway remodelling has been well documented [2].

The pathogenetic mechanism implicated in airway remodelling is not yet well understood. It is possible that the destruction of the epithelial cells leads to release of growth factors, in an attempt to regain the epithelium integrity. These growth factors cause thickening of the reticular basement membrane and proliferation of smooth muscle cells [1]. Specifically, the increase of airway smooth muscle cell (ASMC) number occurring in asthma and in COPD may be, at least partially, caused by growth factors, such as the platelet-derived growth factor (PDGF), the epidermal growth factor (EGF), the insulin-like growth factor-1 (IGF-1), and the basic fibroblast growth factor (bFGF) [3]. PDGF, EGF, IGF-1, and bFGF can activate the mitogen-activated protein kinases (MAPKs) and phosphoinositide 3-kinase (PI3K) signalling pathways and lead to proliferation of smooth muscle cells [4–7]. These growth factors are also related to the inflammation itself, since they can be released by inflammatory cells such as eosinophils and macrophages. They can also be released by epithelial cells or even smooth muscle cells [8, 9]. Many researchers believe that apart from growth factors, other factors that are released during inflammation may as well be involved in the airway remodelling [10–12].

Airway inflammation, both acute and chronic, is mediated by cytokines, such as interleukin (IL-1*β*, IL-5, IL-4, IL-9, IL-10, and IL-13) and tumour necrosis factor alpha (TNF-*α*), and mediators, such as the cysteinyl leukotrienes and isoprostanes, that are increased in asthmatic airways [13]. There is evidence that airway myofibroblasts as well as myocytes perpetuate local airway wall inflammation that leads to structural change [14, 15]. In vitro studies show that TNF-*α* and IL-1*β* induce the secretion of proinflammatory cytokines, chemokines, growth factors, and adhesion molecules and therefore modulate synthetic function of ASMC [16, 17] and alter the responsiveness to contractile agonists (bradykinin, carbachol) and/or relaxant factors (isoproterenol) [18, 19].

The increase in smooth muscle occurring in airway remodelling has been recognized as a major factor that affects airway responsiveness and consequently the severity of airway obstruction and airflow limitation observed in the above diseases [2]. Therefore, the in-depth understanding of the impact of growth factors or cytokines on airway smooth muscle mass is of great interest, since an approach aimed at decreasing airway smooth muscle mass may offer new therapeutic targets in asthma and COPD.

Cultured ASMCs are characterised by plasticity, namely, they are not terminally differentiated but have the ability to switch between different phenotypes. These phenotypic changes of ASMCs, from “contractile” to “synthetic” or “hypercontractile”, are implicated in the pathology of airway diseases. The distinction between the different ASMC phenotypes is based on the different expression of proteins implicated in the contraction mechanism, as well as cell proliferation and protein synthesis. ASMC cultures are a mixed population of cells exhibiting different phenotypes. Although “synthetic” ASMCs appear to have the capability to proliferate, a variety of effects of mitogenic stimuli on these cells are observed [4–6].

The aim of the current study was to clarify the mitogenic effect of the cytokines IL-1*β* or TNF-*α* and of the growth factors bFGF, TGF, or PDGF on ASMC proliferation. In addition we studied the effect of these growth factors on the cell phenotype, as well as the ability of airway smooth muscle to contract. In order to investigate the reliance of the proliferative effect of the above agents with the type of ASMC, experiments were carried out in primary cultures of adult rabbit tracheal ASMCs and human bronchial ASMCs, which are obtained from the upper and the lower parts of the airways.

## 2. Methods

### 2.1. Animals

Rabbits were maintained in individual cages under a controlled environment consisting of a 12 h light-dark cycle and ambient temperature of 22°C and were provided with food and water before use for the study. Animals were treated in compliance with ethical and institutional guidelines. 

### 2.2. ASMCs Isolation and Culture

The isolation and culture of rabbit tracheal ASMC were done as previously described [20, 21]. Briefly, tracheal muscle was epithelium denuded, dissected from cartilage, and washed in low Ca^2+^ Krebs solution (139 mM NaCl, 5.4 mM KCl, 1.47 mM MgSO_4_, 11 mM glucose, 1.47 mM KH_2_PO_4_, 2.8 mM Na_2_HPO_4_, 1.4 mM NaHCO_3_, and 0.2 mM CaCl_2_). Tracheal smooth muscle was digested in 2 mL of low Ca^2+^ Krebs solution containing 0.25% bovine serum albumin (BSA), 2 mg/mL collagenase I, and 10 U/mL elastase IV, for 30 min at 37°C with vigorous shaking. Then it was washed in low Ca^2+^ Krebs solution, centrifuged (1000 rpm for 10 min) and incubated in low Ca^2+^ Krebs solution containing 0.25% bovine serum albumin (BSA), 1 mg/mL collagenase I, and 20 U/mL elastase IV. Dispersed ASMCs were washed and centrifuged (1000 rpm for 10 min) twice in Dulbecco's modified Eagle's mediufm/Ham/F12 (DMEM/F12) containing 10% FBS, 100 U/mL penicillin, and 100 *μ*g/mL streptomycin. The isolated ASMCs were placed in culture flasks and grown at 37°C in a humified incubator under 5% CO_2_. 

Human bronchial ASMCs (cc2576) were purchased from Cambrex-Lonza (Lonza Group Ltd, Basel, Switzerland.).

Human bronchial and rabbit tracheal ASMCs were characterised by immunofluorescence with the monoclonal antibody A104 (Sigma-Aldrich Chemie) against smooth muscle *α*-actin.

### 2.3. Cell Culture Treatments

Cells were trypsinised, counted, and seeded into cell culture plates. They were allowed to adhere overnight and incubated in DMEM/F12 containing 100 U/mL penicillin and 100 *μ*g/mL streptomycin for 72 hours, in order to become synchronized. ASMCs were then exposed to the cytokines IL-1*β* (15 ng/mL) or TNF-*α* (1 ng/mL) and the growth factors TGF (20 ng/mL), bFGF (10 ng/mL), or PDGF (25 ng/mL) for 48–72 h. Control cells remained in DMEM/F12 and cells used as positive control for proliferation were incubated with 10% FBS. Cells were pretreated with the MAPK pathway inhibitor PD89005 (100 *μ*M) for 1h and with the PI3K pathway inhibitor LY294002 (20 *μ*M) for 15 min.

### 2.4. Measurement of Proliferation

Proliferation of cultured ASMC was estimated using the Cell Titer 96 AQ_ueous_ One Solution Assay (Promega) method [22, 23] and the methyl-[^3^H]thymidine incorporation method [24].

- *Cell Titer 96 AQ *_*ueous*_ *One Solution Assay (Promega) method* is a colorimetric method based on metabolic reduction of the yellow tetrazolium dye MTS to purple formazan by the action of mitochondrial succinyl dehydrogenase in living cells. The absorbance of the MTS formazan reduction product was measured at 490 nm with a reference at 630 nm in an ELISA plate reader. The measured OD (optical density) is analogous to the cell number in the well, since there is a linear response between the measured OD and cell number (data not shown).
- *The methyl-[* ^3^ *H]thymidine incorporation method* is based on the incorporation of [^3^H]thymidine in DNA during replication in proliferating cells. The methyl-[^3^H]thymidine was added in the culture medium during the last 18 hours of incubation. The counts per minute (cpm) of the radioactive DNA were counted using a Wallac scintillation counter. Cells incubated in serum free medium (nonproliferative cells) were used as negative control in each experiment. The proliferative capability of cells was evaluated by cell incubation in 10% FBS containing medium.

In the methyl-[^3^H]thymidine incorporation method the point of 48 h of incubation was chosen, since ASMCs appear to have a 48–52 h division time.

### 2.5. Western Blot Analysis

Western blot analysis was performed as previously described [25]. Cells were lysed in 20 mM Tris-Cl pH 8.0, 150 mM NaCl, 1% Triton X-100, 1 mM DTT, and 100 *μ*g/mL phenylmethylsulfonyl fluoride (PMSF). Total cell extracts were cleared by centrifugation (10000 g for 20 min at 4°C). 40 *μ*g of protein was analyzed in 10% sodium dodecyl sulphate-polyacrylamide electrophoresis gel (SDS-PAGE) and transferred to a nitrocellulose membrane. Western blot analysis was performed with anti-human smooth muscle *β*-actin mouse monoclonal antibody (1 : 5000, Cell Signalling), anti-human phospho-Akt rabbit polyclonal antibody (1 : 1000 Cell Signaling), anti-human phospho-p42/44 rabbit polyclonal antibody (1 : 1000 Cell Signaling), and anti-human phospho-p38 rabbit polyclonal antibody (1 : 1000, Cell Signaling). Membranes were then incubated with horseradish-conjugated anti-rabbit IgG (1 : 3000) or anti-mouse IgG (1 : 3000), followed by enhanced chemiluminescence (ECL).

### 2.6. Contractility Studies

Tracheal strips were isolated from adult rabbits [20] and, after epithelium removal, were incubated in DMEM-F-12, with or without bFGF for 3 days. The strips were stretched manually to 1 g of resting tension and allowed to equilibrate for ≥60 min. Contractions were induced by 10^−9^–10^−4^ M carbachol and changes in tension recorded on a force displacement transducer (Grass FT03C, Astro Med) and displayed via an oscillograph recorder (model 7400, Grass). Values are expressed as tension in grams per tissue cross-section (in mm^2^).

### 2.7. Indirect Immunofluorescence

ASMCs, grown on coverslips, were incubated with anti-SM *α*-actin mouse monoclonal antibody (A104, Sigma) following incubation with a secondary CY3-conjugated anti-mouse IgG antibody and mounted on Vectrashield containing DAPI for DNA staining. The number of cells expressing *α*-actin was counted and expressed as percentage of the total number of cells stained with DAPI.

### 2.8. Statistical Analysis

In cell proliferation experiments each point was performed in triplicate and the values presented are the mean of independent experiments. All data are expressed as means ± standard error of the mean (SEM) and *N* refers to the number of experiments. Differences between means were analyzed by one-way ANOVA with Bonferroni's posttest or unpaired *t*-test with statistically significant differences between groups being determined by Mann-Whitney test. A comparison is considered significant when *P* < 0.05. The statistical analysis was performed using GraphPad Image (GraphPad Software, San Diego, CA, USA). Analysis of the western blotting images was conducted with the use of Photoshop 7.0 (http://www.adobe.com/products/photoshopfamily/), and results were expressed as intensity values.

## 3. Results

### 3.1. The Effect of the Proinflammatory Cytokines Interleukin 1*β* (IL-1*β*) and Tumor Necrosis Factor *α* (TNF-*α*) on ASMC Proliferation

**Figure 1:**
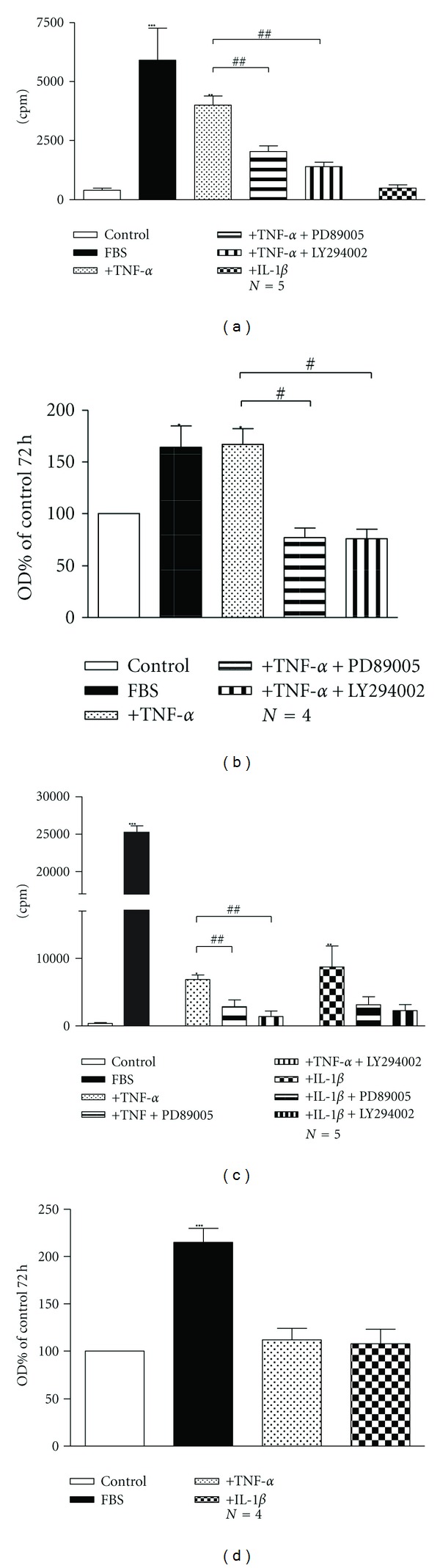
The effect of TNF-*α* (1 ng/mL) and IL-1*β* (15 ng/mL) on cell proliferation of human bronchial ((a) and (b)) and rabbit tracheal ((c) and (d)) ASMC. Methyl-[^3^H]thymidine incorporation after 48 h of incubation of ASMC ((a) and (c)) and cell number after 72 h of incubation ((b) and (d)). Where indicated, cells were treated with the MAPK pathway inhibitor, PD89005 (100 *μ*M), or the PI3K pathway inhibitor, LY294002 (20 *μ*M). Data are presented as mean ± SEM, and *N* refers to the number of independent experiments. *P < 0.05, **P < 0.01, ***P < 0.001 compared to control (one-way ANOVA with statistically significant differences between groups being determined by Bonferroni's posttest) and ^#^ *P* < 0.05 and ^##^ *P* < 0.01 compared to the effect of TNF-*α* alone (unpaired *t*-test with statistically significant differences between groups being determined by Mann-Whitney test).

Incubation of human bronchial ASMCs with TNF-*α* ( 1 ng/mL) increased significantly methyl-[^3^H]thymidine incorporation after 48 h (*P* < 0.01, Figure 1(a)), accompanied by an increase in cell number after 72 h (*P* < 0.05; Figure 1(b)). Pretreatment of human ASMCs with the MAPK pathway inhibitor PD89005 (100 *μ*M) and the PI3K pathway inhibitor LY294002 (20 *μ*M) reduced the proliferative effect of TNF-*α* on human ASMCs (Figure 1). In contrast to TNF-*α*, IL-1*β* had no effect on methyl-[^3^H]thymidine incorporation in human ASMCs (Figure 1(a)).

**Figure 2:**
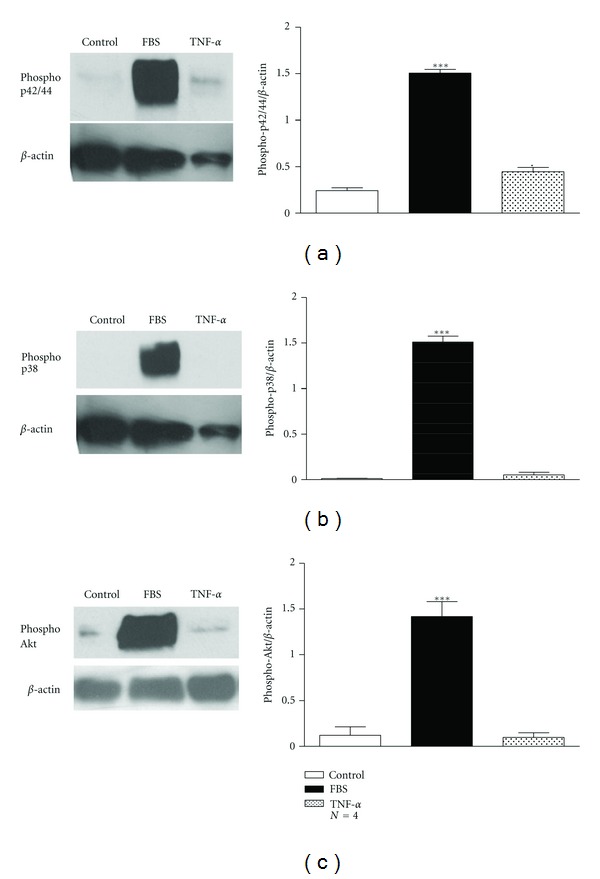
Induction of the MAPK and PI3K signaling in human bronchial ASMCs incubated with TNF-*α*. *Left: *Western blot analysis with (a) antiphospho-p42/44, (b) antiphospho-p38, and (c) antiphospho-Akt and anti-*β*-actin antibodies in total protein cell extracts from human bronchial smooth muscle cells incubated for 4 h with TNF-*α* (1 ng/mL). *Right: *mean fold expression of phospho-p42/44, phospho-p38 or phospho-Akt normalized to *β*-actin. Data are presented as mean ± SEM, and *N* refers to the number of independent experiments. *P < 0.05 and ***P < 0.001 compared to control (one-way ANOVA with statistically significant differences between groups being determined by Bonferroni's posttest).

**Figure 3:**
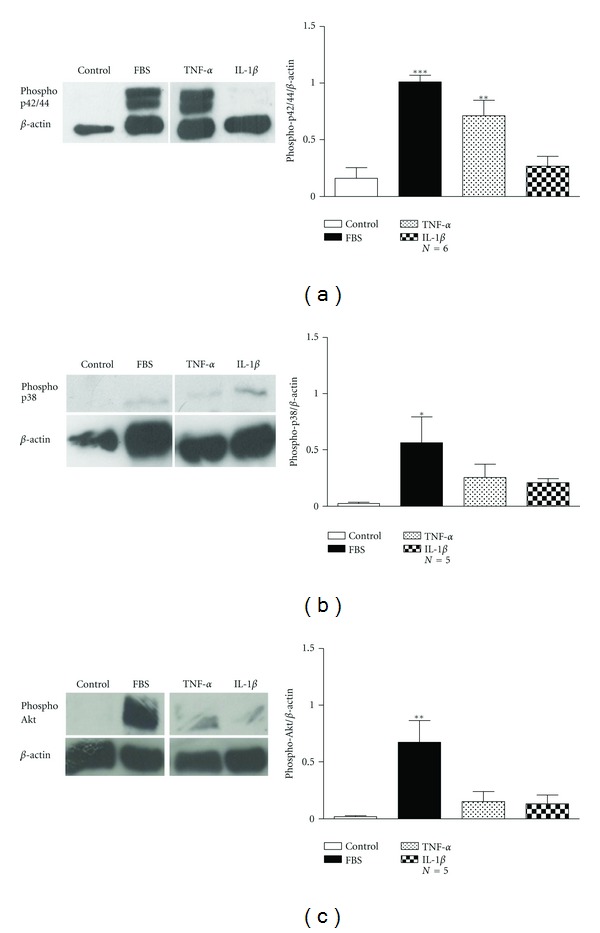
Induction of the MAPK and PI3K signalling in rabbit tracheal ASMCs incubated with TNF-*α* or IL-1*β*. *Left*: western blot analysis with (a) antiphospho-p42/44, (b) antiphospho-p38, and (c) antiphospho-Akt and anti-*β*-actin antibodies in total protein cell extracts from rabbit tracheal smooth muscle cells incubated for 4 h with TNF-*α* (1 ng/mL) or IL-1*β* (15 ng/mL). *Right*: mean fold expression of (a) phospho-p42/44, (b) phospho-p38, or (c) phospho-Akt to *β*-actin. Data are presented as mean ± SEM, and *N* refers to the number of independent experiments. *P < 0.05, **P < 0.01, and ***P < 0.001 compared to control (one-way ANOVA with statistically significant differences between groups being determined by Bonferroni's posttest).

Regarding rabbit tracheal ASMCs, both TNF-*α* (1 ng/mL) and IL-1*β* (15 ng/mL) increased significantly methyl-[^3^H]thymidine incorporation, after 48 h of incubation (*P* < 0.05, Figure 1(c)). The effect of TNF-*α* and IL-1*β* on methyl-[^3^H]thymidine incorporation was reversed by the MAPK pathway inhibitor PD89005 (100 *μ*M) and the PI3K pathway inhibitor LY294002 (20 *μ*M) (Figure 1(c)). On the other hand neither TNF-*α* nor IL-1*β* affected cell number (Figure 1(d)). The incubation of ASMCs with TNF-*α* resulted in the activation of p42/p44 MAPK as shown by the increase of the phosphorylated p42/44 protein, but not of the p38 MAPK or PI3K signalling pathway, in total extracts prepared from human bronchial ASMCs (Figure 2). In rabbit tracheal ASMCs, TNF-*α* activated significantly only the p42/p44 signalling pathway (Figure 3) while IL-1*β* did not reveal a significant activation of the p42/p44 or p38 MAPK or PI3K signalling pathway (Figure 3).

### 3.2. The Effect of Transforming Growth Factor (TGF), Basic Fibroblast Growth Factor (bFGF), or Platelet-Derived Growth Factor (PDGF) on ASMC Proliferation

**Figure 4:**
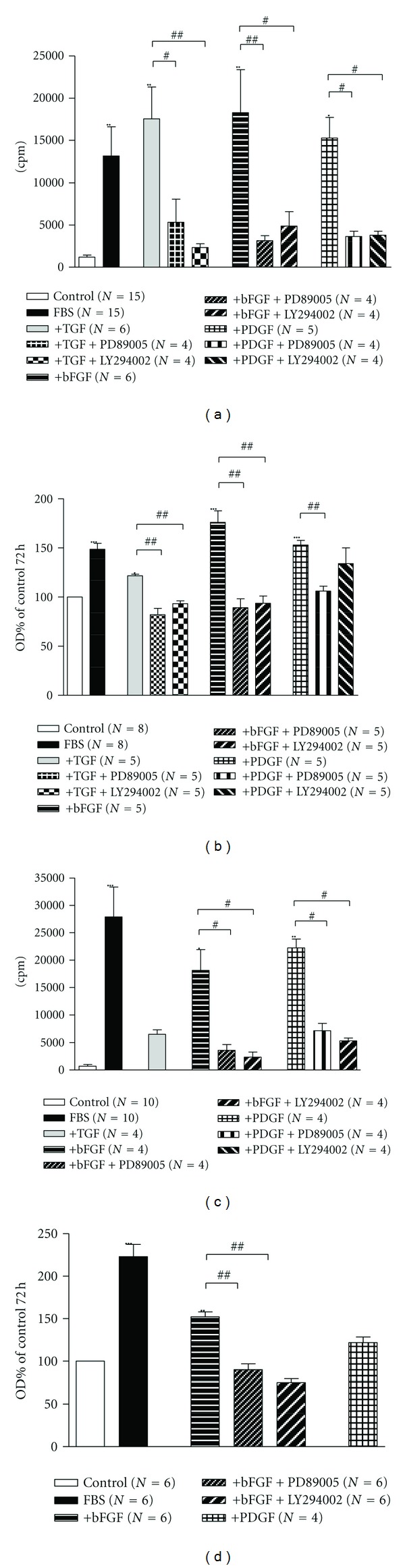
The effect of bFGF (10 ng/mL), TGF (20 ng/mL), and PDGF (25 ng/mL) on cell proliferation of human bronchial ((a) and (b)) and rabbit tracheal ((c) and (d)) ASMC. Methyl-[^3^H]thymidine incorporation after 48 h of incubation of ASMC ((a) and (c)) and cell number after 72 h of incubation ((b) and (d)). Where indicated, cells were treated with the MAPK pathway inhibitor, PD89005 (100 *μ*M) or the PI3K pathway inhibitor, LY294002 (20 *μ*M). Data are presented as mean ± SEM, and *N* refers to the number of independent experiments. *P < 0.05, **P < 0.01, ***P < 0.001 compared to control (one-way ANOVA with statistically significant differences between groups being determined by Bonferroni's posttest) and ^#^ *P* < 0.05 and ^##^ *P* < 0.01 compared to the effect of bFGF, TGF, or PDGF alone (unpaired *t*-test with statistically significant differences between groups being determined by Mann-Whitney test).

Incubation of human bronchial ASMCs with TGF (20 ng/mL), bFGF (10 ng/mL), or PDGF (25 ng/mL) increased significantly (*P* < 0.05) methyl-[^3^H]thymidine incorporation (Figure 4(a)) and cell number (Figure 4(b)). Preincubation of ASMCs with PD89005 (100 *μ*M) or LY294002 (20 *μ*M) reduced significantly the mitogenic effect of all growth factors on human bronchial ASMCs (Figure 4).

The incubation of rabbit tracheal ASMCs with bFGF (10 ng/mL) or PDGF (25 ng/mL) for 48 h increased significantly (*P* < 0.05) methyl-[^3^H]thymidine incorporation (Figure 4(c)), while TGF (20 ng/mL) had no effect. However, only in the presence of bFGF, we detected a significant increase in cell number (*P* < 0.01), estimated with Cell Titer 96 Aqueous Method that was again sensitive to MAPK and PI3K inhibitors (Figure 4(d)). 

**Figure 5:**
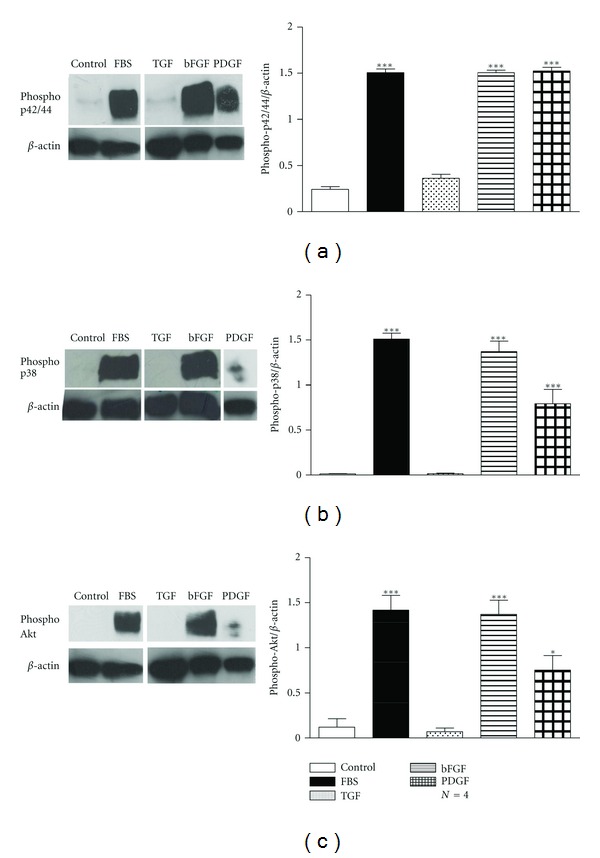
Induction of the MAPK and PI3K signalling in human bronchial ASMCs incubated with bFGF, TGF, or PDGF. *Left: *western blot analysis with (a) antiphospho-p42/44, (b) antiphospho-p38, and (c) antiphospho-Akt and anti-*β*-actin antibodies in total protein cell extracts from human bronchial smooth muscle cells incubated for 4 h with bFGF (10 ng/mL), TGF (20 ng/mL) or PDGF (25 ng/mL). *Right: *mean fold expression of phospho-p42/44, phospho-p38, or phospho-Akt normalized to *β*-actin. Data are presented as mean ± SEM, and *N* refers to the number of independent experiments. *P < 0.05 and ***P < 0.001 compared to control (one-way ANOVA with statistically significant differences between groups being determined by Bonferroni's posttest).

Western blot analysis with antiphospho-Akt, antiphospho-p42/44 or antiphospho-p38 antibodies of total protein cell extracts from human bronchial ASMCs incubated for 4 h with the growth factors, revealed that bFGF (10 ng/mL) or PDGF (25 ng/mL) activated the p42/44, p38, and PI3K signalling pathway (Figure 5), while TGF (20 ng/mL) seems not to affect significantly the activation of the above signalling pathways. 

**Figure 6:**
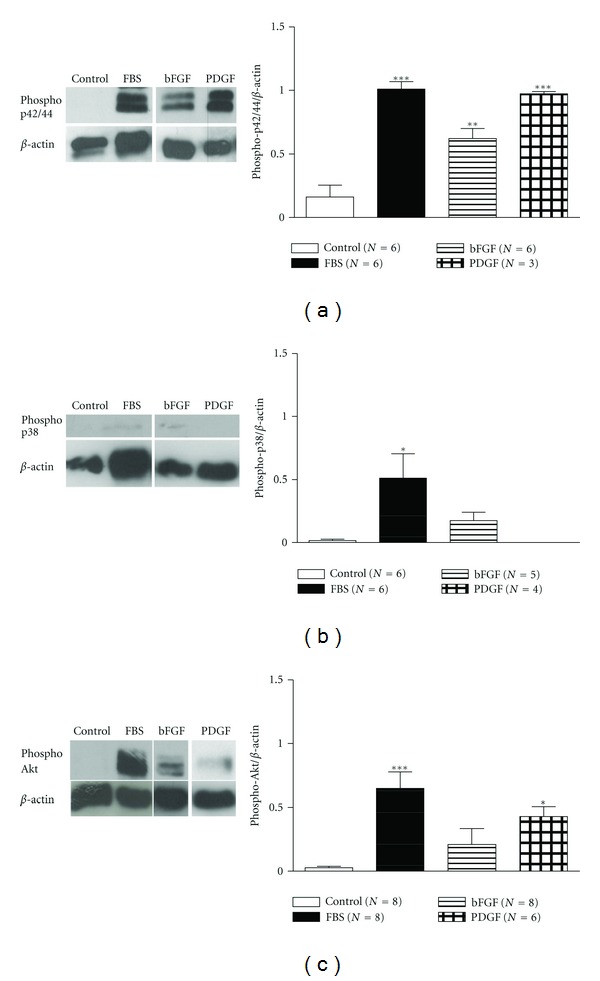
Induction of the MAPK and PI3K signalling in rabbit tracheal ASMCs incubated with bFGF and PDGF. *Left: *western blot analysis with (a) antiphospho-p42/44, (b) antiphospho-p38, and (c) antiphospho-Akt and anti-*β*-actin antibodies in total protein cell extracts from rabbit tracheal smooth muscle cells incubated for 4 h with bFGF (10 ng/mL) or PDGF (25 ng/mL). *Right: *mean fold expression of phospho-p42/44, phospho-p38, or phospho-Akt normalized to *β*-actin. Data are presented as mean ± SEM, and *N* refers to the number of independent experiments. *P < 0.05, **P < 0.01, and ***P < 0.001 compared to control (one-way ANOVA with statistically significant differences between groups being determined by Bonferroni's posttest).

In rabbit tracheal ASMCs, bFGF (10 ng/mL) or PDGF (25 ng/mL) activated the p42/44 signalling pathway, while only PDGF activated the PI3K signalling pathway (Figure 6).

### 3.3. The Effect of Basic Fibroblast Growth Factor (bFGF) on the Responsiveness of Rabbit Tracheal Strips to Carbachol

**Figure 7:**
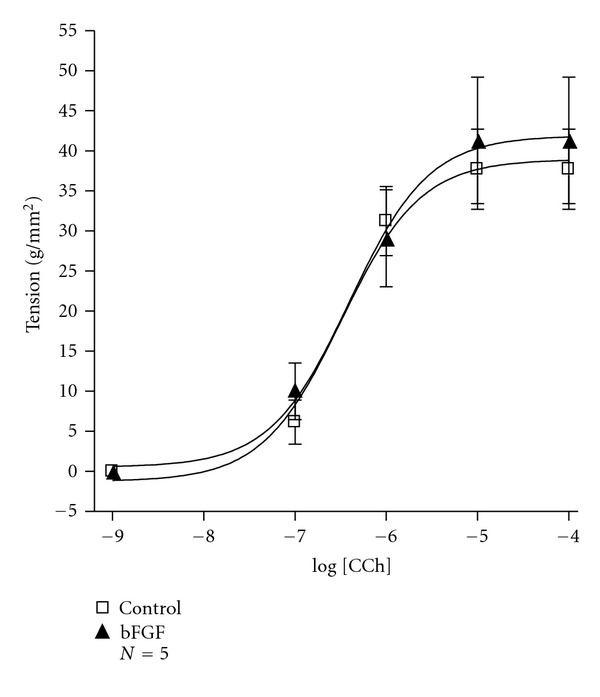
Dose-response curve of rabbit tracheal strips to carbachol (CCh; 10^−9^ M–10^−4^ M) after 72 h of incubation in DMEM-F-12 containing bFGF (10 ng/mL). Data are presented as mean ± SEM, and *N* refers to the number of independent experiments.

Since bFGF (10 ng/mL) increased rabbit tracheal ASMC number after 72 h of incubation (Figure 4) we performed contractility studies with rabbit tracheal strips to investigate the possible effect of bFGF on their responsiveness to carbachol. These experiments showed that the incubation of tracheal strips with bFGF (10 ng/mL) for 72 h did not alter their responsiveness to carbachol (Figure 7). Namely, maximal contraction obtained from tracheal strips incubated in the absence and the presence of bFGF was 38.95 ± 0.53 and 41.90 ± 0.32 g/mm^2^ (*N* = 5), respectively.

### 3.4. The Effect of Transforming Growth Factor (TGF), Basic Fibroblast Growth Factor (bFGF), or Platelet-Derived Growth Factor (PDGF) on ASMC Phenotype

**Figure 8:**
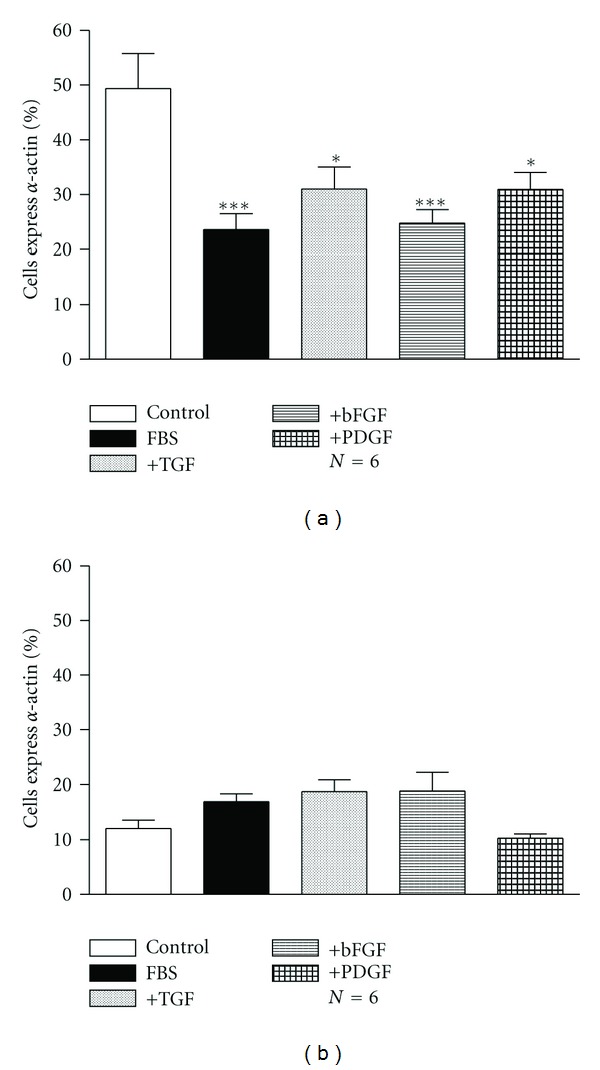
Percentage of (a) human bronchial and (b) rabbit tracheal ASMCs expressing *α*-actin, estimated by indirect immunofluorescence with an anti-*α*-actin antibody after incubation for 72 h with bFGF (10 ng/mL), TGF (20 ng/mL), or PDGF (25 ng/mL). Data are presented as mean ± SEM, and *N* refers to the number of independent experiments. *P < 0.05 and ***P < 0.001 compared to control (one-way ANOVA with statistically significant differences between groups being determined by Bonferroni's posttest).

The incubation of human bronchial ASMCs with TGF (20 ng/mL), bFGF (10 ng/mL), or PDGF (25 ng/mL) decreased the percentage of cells expressing *α*-actin (Figure 8(a)) (*P* < 0.05), while the effect of these growth factors was not statistically significant in rabbit tracheal ASMCs (Figure 8(b)).

## 4. Discussion

A common feature in inflammatory airway diseases, like asthma and COPD, is airway smooth muscle thickening [1]. In the present study we first investigated the potential effect of the proinflammatory cytokines TNF-*α* and IL-1*β*, which are chronically present in asthmatic airways. It is generally believed that they modulate ASM function through binding to specific receptors expressed on the ASMC surface. Native tracheal tissues express TNFR-1 and TNFR-2 [26], although TNFR-1 appears to be the receptor regulating the induction of IL-6, ICAM-1, and RANTES expression by TNF-*α* in human ASMC [27].

We have found that the effect of TNF-*α* and IL-1*β* on ASMC proliferation varied. Only TNF-*α*, but not IL-1*β*, induced an increase in [^3^H]thymidine incorporation and cell number of human bronchial ASMCs, while both cytokines enhanced [^3^H]thymidine incorporation in rabbit tracheal ASMCs (Figure 1). All the effects on cell proliferation were diminished by the use of PI3K or MAPK pathway inhibitors (Figure 1), confirming the essential role of these pathways in ASMC proliferation, although only TNF-*α* had a significant effect on p22/p44 MAPK signalling pathway activation in both human and rabbit ASMCs (Figures 2 and 3).

The variability of the TNF-*α* and IL-1*β* proliferative effect has been observed also in other studies. Some report a positive mitogenic effect of TNF-*α* or IL-1*β* on ASMC, which depends on the activation of the p42/p44 MAPK pathway [28–30]. In contrast, others claim that TNF-*α* or IL-1*β* fails to induce proliferation on its own [31–33] and inhibit proliferation induced by growth factors [34]. These, at first view contradictory, results can be explained by the fact that cytokines, along with the activation of the promitogenic MAPK pathway, stimulate the antimitogenic IFN*β* [35] and cAMP-dependent protein kinase [34]. In addition, glucocorticoids have been shown to modulate the proliferative effect of proinflammatory cytokines on ASM, in a negative or positive way, depending on the presence of other growth factors [34, 36]. Collectively, these results indicate that the regulation of ASMC proliferation by proinflammatory cytokines is rather complex and partly depends on their antagonistic and synergistic relationship with growth factors and glucocorticoids.

Growth factors, which are released during inflammation in chronic airway diseases, in an attempt to regain epithelium integrity, also lead to the proliferation of ASMC [1]. They are released from a variety of both inflammatory (eosinophils and macrophages) and structural cells, such as epithelial and even human bronchial ASMCs [3, 8]. There are reports of bFGF, TGF, and PDGF inducing ASMC number increase in asthma or COPD [3, 37]. 

We investigated the effect of these growth factors on the proliferation of both human bronchial and rabbit tracheal ASMCs. The mitogenic effect of the above growth factors varied depending on cell type. Namely, bFGF had a mitogenic effect on both human and rabbit ASMCs (Figure 4) as it increased both DNA synthesis and cell number. TGF increased cell proliferation only of human ASMC without any effect on rabbit ASMCs (Figure 4). Last, PDGF increased [^3^H]thymidine incorporation in both human bronchial and rabbit tracheal ASMCs but affected the cell number only of human bronchial ASMCs (Figure 4). These results indicate that human bronchial ASMCs are more sensitive to the proliferative effect of growth factors compared to rabbit tracheal cells. 

Growth factors are known to activate signalling pathways that lead cells to proliferation [4–7]. In our experiments the effect of the growth factors, in both cell types, was reduced by the inhibitors of the MAPK and PI3K pathway. Our findings are in compliance with data showing that bFGF induces proliferation of human bronchial ASMCs, by activation of p38 MAPK signalling pathway [38], alone or in the presence of TGF or PDGF [11]. 

On the other hand the role of TGF is controversial. There are reports on the induction of fibroblasts, myofibroblast or SMC proliferation [39] but this induction depends on the confluence of the culture and the concentration of the growth factor. Specifically, TGF stimulates proliferation of confluent vascular smooth muscle cells and ASMCs, but inhibits the proliferation of the same cells when they are subconfluent [40–43]. Also in a low dose TGF-*β*1 stimulates proliferation of fibroblasts, chondrocytes, and arterial smooth muscle cells, but a high dose of TGF-*β*1 inhibits the proliferation of the same cells [39–44]. The induction of proliferation of bovine tracheal ASMCs in the presence of TGF is accompanied by activation of the MAPK pathway [37, 45].

PDGF has been reported to act as a mitogenic stimulus in human coronary arterial SMCs [46], as well as human tracheal ASMCs [10].

Our experiment demonstrates that bFGF has a clear mitogenic effect on rabbit tracheal ASMCs (Figure 4). We also investigated if this effect may modulate the responsiveness of the whole tissue, specifically tracheal strips, to carbachol. Therefore we performed contractility studies on epithelium-denuded tracheal strips incubated for 3 days in the presence of bFGF. These experiments demonstrated that bFGF did not alter the responsiveness of tracheal strips to carbachol (Figure 7). These results suggest that ASMC behaviour may differ between cell culture and tissue, or structural changes in the cells might lead to phenotype alteration. 

It is well documented that ASMCs have phenotype plasticity, therefore the ability to alter their phenotype, by changing the expression of contractile proteins and their responsiveness to stimuli that cause cell contraction [12]. In order to investigate the possible effect of growth factors on ASMC phenotype we measured the percentage of cells that express *α*-actin in the presence of growth factors. Incubation for 72 h with TGF, bFGF, and PDGF decreased the percentage of human ASMCs expressing *α*-actin with no effect on rabbit cells phenotype (Figure 8). These data suggest that although growth factors stimulate ASMC proliferation, the possible shift from the contractile to the proliferative cell phenotype may be the reason we do not observe an analogous increase of airway smooth muscle tissue responsiveness to contractile agents.

In conclusions, the results from this study demonstrate mechanisms of ASMC proliferation in response to factors that are released in the airway during chronic airway diseases, where remodelling of the airway wall increases the severity of the symptoms and therefore could be of clinical significance. Inflammatory (TNF-*α*) and growth factors (TGF, bFGF, and PDGF) that are usually released during inflammation in chronic airway diseases, such as asthma and COPD, have a mitogenic effect and they may be implicated in airway remodelling. Their effect is mediated via the PI3K, as well as the p38 and/or the p42/44 MAPK, signalling pathways. Moreover, the effect of different cytokines and growth factors on ASMC appears to depend on the specific cell type, with bronchial ASMCs being more prone to proliferation than tracheal ASMCs. Finally, growth factors may cause a switch of the ASMC to the proliferative phenotype that may hinder an increase of ASM responsiveness to contractile agents comparable to the increase of ASMC mass.
